# Bidirectional pharmacokinetic drug interactions between olaparib and metformin

**DOI:** 10.1007/s00280-023-04591-y

**Published:** 2023-10-10

**Authors:** Joanna Stanisławiak-Rudowicz, Agnieszka Karbownik, Danuta Szkutnik-Fiedler, Filip Otto, Tomasz Grabowski, Anna Wolc, Edmund Grześkowiak, Edyta Szałek

**Affiliations:** 1 Department of Clinical Pharmacy and Biopharmacy, Poznań University of Medical Sciences, Rokietnicka 3, 60-806 Poznań, Poland; 2 Poznań University Clinical Hospital, Szamarzewskiego 84/86, 60-569 Poznań, Poland; 3 Department of Inorganic Chemistry, Faculty of Pharmacy, Medical University of Gdańsk, M. Skłodowskiej-Curie 3a, 80-210 Gdańsk, Poland; 4 Department of Animal Science, Iowa State University, 239E Kildee Hall, Ames, IA 50011 USA; 5 Hy-Line International, 2583 240th Street, Dallas Center, IA 50063 USA

**Keywords:** Olaparib, Metformin, Pharmacokinetics, Drug–drug interactions

## Abstract

**Objective:**

Olaparib is a PARP (poly-ADP-ribose polymerase) inhibitor used for maintenance therapy in BRCA-mutated cancers. Metformin is a first-choice drug used in the treatment of type 2 diabetes. Both drugs are commonly co-administered to oncologic patients with add-on type 2 diabetes mellitus. Olaparib is metabolized by the CYP3A4 enzyme, which may be inhibited by metformin through the Pregnane X Receptor*. *In vitro studies have shown that olaparib inhibits the following metformin transporters: OCT1, MATE1, and MATE2K. The aim of the study was to assess the influence of ‘the perpetrator drug’ on the pharmacokinetic (PK) parameters of ‘the victim drug’ after a single dose. To evaluate the effect, the AUC_0→∞_ (area under the curve) ratio was determined (the ratio between AUC_0→∞_ in the presence of the perpetrator and AUC_0→∞_ without the presence of the perpetrator).

**Methods:**

Male Wistar rats were assigned to three groups (eight animals in each group), which were orally administered: metformin and olaparib (I_MET+OLA_), *vehiculum* with metformin (II_MET_), and *vehiculum* with olaparib (III_OLA_). Blood samples were collected after 24 h. HPLC was applied to measure the concentrations of olaparib and metformin. The PK parameters were calculated in a non-compartmental model.

**Results:**

Metformin did not affect the olaparib PK parameters. The AUC_0→∞_ I_MET+OLA_/III_OLA_ ratio was 0.99. Olaparib significantly increased the metformin *C*_max_ (by 177.8%), AUC_0→t_ (by 159.8%), and AUC_0→∞_ (by 74.1%). The AUC_0→∞_ I_MET+OLA_/II_MET_ ratio was 1.74.

**Conclusions:**

A single dose of metformin did not affect the PK parameters of olaparib, nor did it inhibit the olaparib metabolism, but olaparib significantly changed the metformin pharmacokinetics, which may be of clinical importance.

**Supplementary Information:**

The online version contains supplementary material available at 10.1007/s00280-023-04591-y.

## Introduction

Olaparib is a medication used for maintenance therapy in BRCA-mutated cancers. It inhibits poly-ADP ribose polymerase (PARP) – an enzyme involved in DNA repair. This drug is mainly indicated for ovarian cancer, fallopian tube cancer, peritoneal cancer, pancreatic cancer, and prostate cancer with hereditary or somatic BRCA1 or BRCA2 mutation [1, 2]. It was first approved as a single agent by the European Medicines Agency (EMA) in the European Union and by the Food and Drug Administration (FDA) in the United States in 2014. It was first approved as a maintenance therapy for recurrent high-grade platinum-sensitive ovarian cancer in BRCA1/2-mutated patients. The maintenance therapy should be started 8 weeks after the last course of platinum-based chemotherapy if there was a partial or complete response to the treatment [3, 4]. Olaparib is an oral drug – 50-mg hard capsules which were mainly changed to film-coated tablets with a starting dose of 300 mg twice a day, in combination with bevacizumab are recommended in the treatment of high-risk ovarian cancer. The main side effects of olaparib are: anemia, leukopenia, nausea, vomiting, and fatigue [1, 4].

Metformin, which is a biguanide derivative, is a first-line drug applied to patients with type 2 diabetes or prediabetes. It is assumed that the antihyperglycemic effect of metformin is achieved through the inhibition of the mitochondrial complex I (NADH + H^+^ dehydrogenase), which changes not only the NAD^+^/NADH + H^+^ ratio, but also the AMP/ATP ratio. An increase in the AMP/ATP ratio is the driving force for AMP-activated protein kinase (AMPK – 5’-adenosinemonophosphate-activated protein kinase). AMPK is also significant in the activation of the p53 protein, which is colloquially known as ‘the guardian of the genome’. AMPK acts as a suppressor in cells exposed to mutations or damage. This leads to their apoptosis when the repair processes fail. AMPK is also associated with other cellular pathways, e.g., STAT, and it regulates the levels of their most important proteins. Research has shown that metformin significantly reduces the levels of p-STAT3 and C-MYC proteins. This decrease is even greater in the presence of a PARP inhibitor. As a result, the proliferative properties of neoplastic cells are suppressed [5]. Apart from that, metformin induces mitochondrial shock and thus causes additional DNA damage by redirecting metabolism in favor of the formation of reactive oxygen species [6]. Metformin is also considered to be involved in the inhibition of genes encoding the CYP3A4 enzyme. For this reason, the metabolism of this enzyme’s substrates, such as olaparib, can be expected to decrease significantly [7].

The affinity of PARP inhibitors for transporters involved in passive transport into and out of cells can be used as a desirable interaction in the pharmacokinetics of drugs (especially olaparib [1] and rucaparib [8]). Many drugs, including those exhibiting cytostatic activity, are substrates for these protein transporters, e.g., oxaliplatin and metformin for OCT1, cisplatin, metformin, and propranolol for OCT2, topotecan and metformin for MATE1, oxaliplatin, topotecan, and metformin for MATE2K [9]. The blockage of the transporter protein responsible for the transport from hepatocytes or into to the renal tubules may cause the accumulation of the drug in the body.

Olaparib is a drug which can inhibit such proteins. When the pathways responsible for the distribution of anticancer drugs in the tissues overlap, the antitumor activity of both drugs increases and there is a risk of more severe adverse reactions. The interaction of metformin with olaparib is an example of the interaction whose pharmacodynamic mechanism has been thoroughly investigated. This interaction is particularly important due to the fact that it is very likely that both drugs may be combined with each other, because type II diabetes, to which metformin is dedicated, poses a significant risk of ovarian cancer [7, 10, 11]. In addition, olaparib raises the blood glucose level by blocking GLUT2 transporters, which increases the fasting glucose level. Therefore, it is very likely that antihyperglycemic treatment will be implemented [12].

**Fig. 1:**
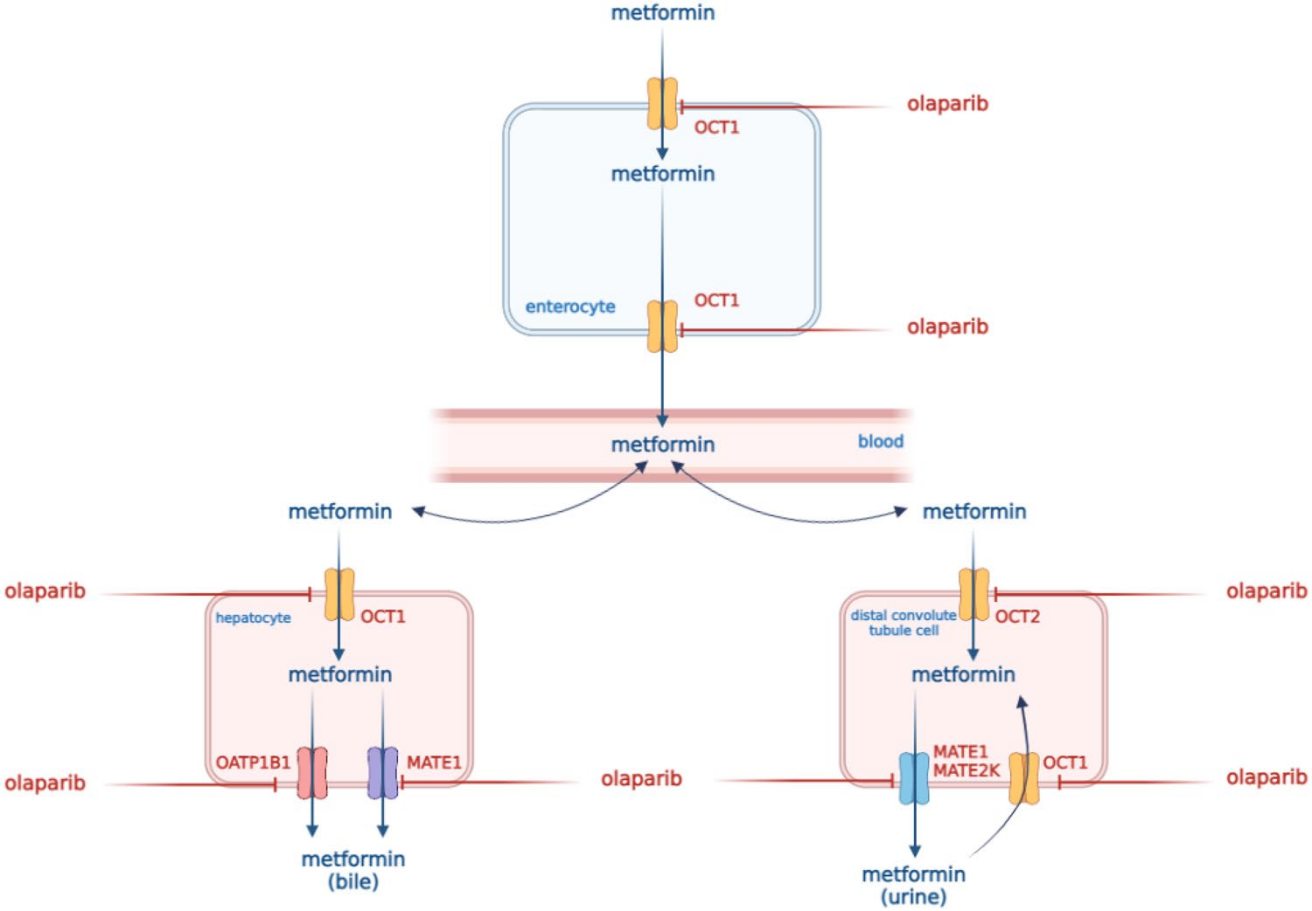
Pharmacokinetic interaction of olaparib with metformin

**Fig. 2:**
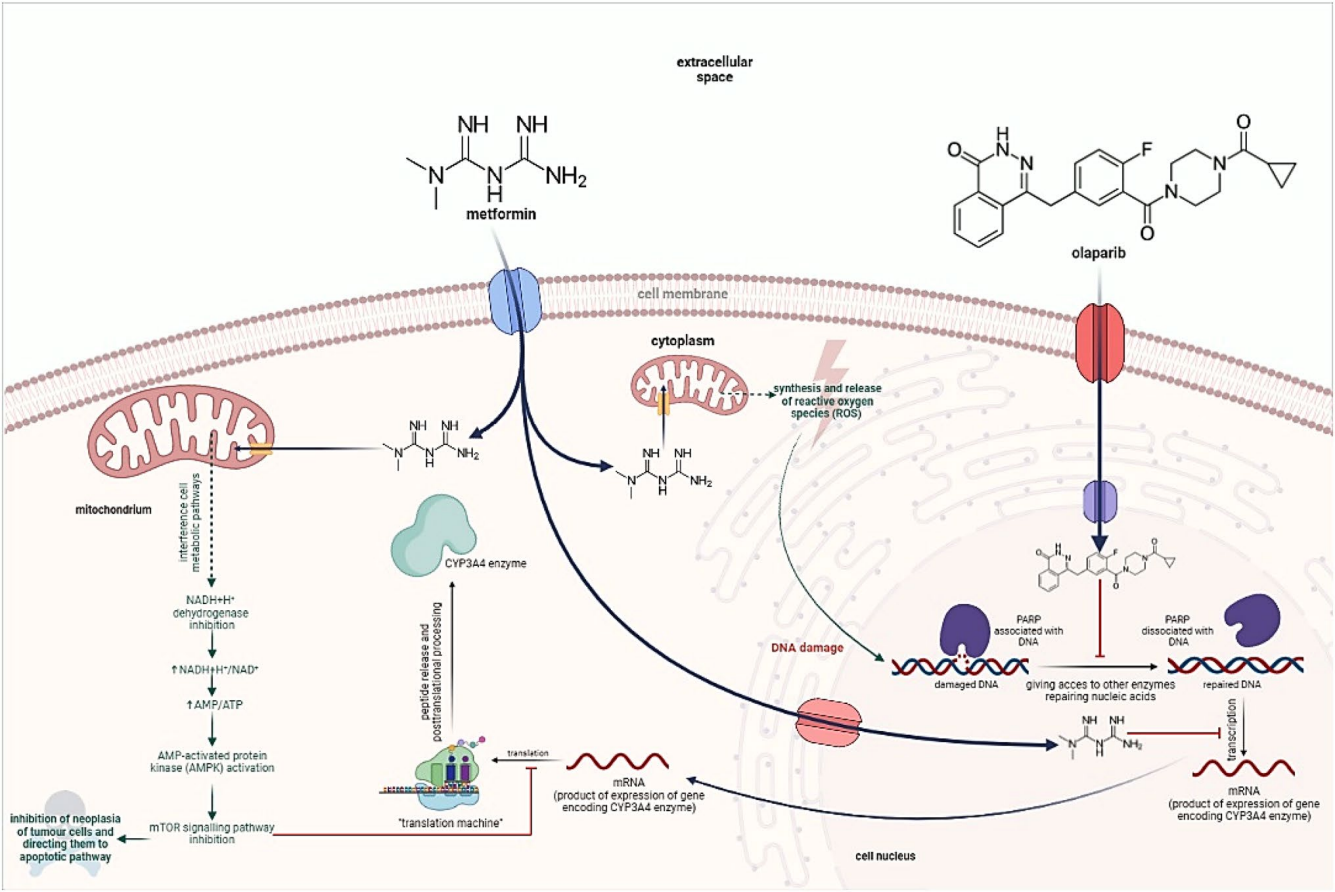
Pharmacodynamic interaction of olaparib with metformin

What is important to manage, metformin and olaparib synergistically inhibit tumor growth by blocking the cell cycle, especially in the S phase, when the synthesis of histone proteins and DNA replication play the most significant role – during the phase specific to PARP inhibitors [13]. The pharmacokinetics and pharmacodynamic interaction of olaparib with metformin are presented in Figs. 1 and 2.

In view of the aforementioned facts, there is a risk of interaction between olaparib and metformin. The aim of our study on animals was to investigate this risk.

## Reagents

Metformin (CAS number 1115–70-4) and olaparib (CAS number 763113–22-0) were purchased from LGC Standards (Łomianki, Poland). Paracetamol (CAS number 103–90-2), olaparib-d4, methanol, acetonitrile, ammonium formate, ammonium acetate, and dimethyl sulfoxide (DMSO) were purchased from Sigma-Aldrich (Poznań, Poland). Water used in the mobile phase was deionized, distilled, and filtered through a Millipore system (Direct Q3, Millipore, USA) before use. Olaparib (Lynparza®, batch number RR214) was purchased from AstraZeneca Pharma Poland Sp. z o.o. (Warsaw, Poland). Metformin (Metformax, batch number 16518316) was purchased from Teva Pharmaceuticals Polska Sp. z o.o. (Warsaw, Poland).

## Animal experiments

All applicable international, national, and/or institutional guidelines for the care and use of animals were followed. The animals were given a standard diet and water ad libitum, and the experimental protocol for this study was approved by the Local Ethics Committee (No. 45/2022, of 27 May 2022), Poznań University of Life Sciences, Department of Animal Physiology and Biochemistry, Wołyńska 35, 60–637 Poznań, Poland. Adult male Wistar rats (weight 380–530 g) were used in the study. The animals were maintained under standard breeding conditions with a 12/12 h light–dark cycle (lights on at 06.00, lights off at 18.00) at constant room temperature (23 ± 2 °C), relative humidity of 55 ± 10% and given ad libitum access to food and water. The animals were allowed to acclimatize for a week before the beginning of the experiments. The rats were divided into three groups. Olaparib was formulated in 10% dimethyl sulfoxide (DMSO) – 90% saline at a single dose of 100 mg/kg [14]. The solution for oral metformin administration was prepared in saline at a single dose of 100 mg/kg [15]. Drugs were orally administered to rats by gavage between 7:00 a.m. and 8:00 a.m. Before administration (0 h) and at different time intervals after the administration, i.e., 0.085, 0.5, 1, 2, 4, 6, 8, 10, and 24 h [16], blood was collected from the tail vein of each rat into 1.5-ml heparinized Eppendorf (EP) tubes. The blood samples were centrifuged at 5000 rpm for 10 min, and the plasma was transferred to new centrifuge tubes and then stored at – 80 °C. However, after performing the tests, it turned out that sampling should be extended in order to achieve lower values of the residual field. Unfortunately, the authors did not indicate the existence of such a problem in their studies.

There were a few cases of high values of the residual area in both groups (> 20%). Therefore, the AUC- or *k*_el_-dependent results of analyses are additionally shown in Table 1B (Table 1A shows *C*_max_, *t*_max_ and AUC_0-t_).

**Table 1:** Plasma pharmacokinetic parameters of metformin (MET) after the oral administration of a single dose of metformin (100 mg/kg b.w.) to the II_MET_ group and metformin + olaparib (100 mg/kg b.w. + 100 mg/kg b.w.) to the I_MET+OLA_ group

Pharmacokinetic parameters	II_MET_ (*n* = 8)	I_MET+OLA_ (*n* = 8)	*p* value I_MET+OLA_ vs. II_MET_	G_mean_ ratio* (90% CI) I_MET+OLA_ vs. II_MET_
Table A				
*C*_max_ (µg/ml)	0.45 ± 0.29 (64.4)	1.25 ± 0.80 (64.2)	0.0117	2.73 (1.56; 4.79)
*t*_max_ (h)	1.09 ± 0.57 (51.7)	2.63 ± 1.85 (70.4)	0.0580	2.26 (1.23; 4.17)
Table B				
AUC_0-t_ (µg × h/ml)	3.83 ± 1.23 (32.1)	9.95 ± 5.09 (32.1)	0.0016	2.45 (1.72; 3.50)
AUC_0-∞_ (µg × h/ml)	7.90 ± 1.93 (24.4)	13.75 ± 5.51 (40.1)	0.0117	1.67 (1.26; 2.22)
*k*_a_ (h^−1^)	0.41 ± 0.25 (62.4)	0.59 ± 0.17 (29.7)	0.1170	1.60 (1.09; 2.37)
*k*_el_ (h^−1^)	0.032 ± 0.014 (42.7)	0.054 ± 0.022 (40.6)	0.0294	1.72 (1.17; 2.53)
*t*_0.5_ (h)	26.54 ± 14.52 (54.7)	14.95 ± 6.04 (40.4)	0.0587	0.58 (0.39; 0.86)
Cl/*F* (l/h)	6.31 ± 1.48 (23.5)	3.57 ± 1.26 (35.34)	0.0014	0.55 (0.41; 0.72)
*V*_d_/*F* (l)	227.66 ± 90.97 (40.0)	79.61 ± 43.04 (54.1)	0.0010	0.32 (0.20; 0.51)

Arithmetic means and standard deviations (SD) are shown with coefficients of variation *CV* (%) in brackets

*C*_*max*_ the maximum plasma concentration; *AUC*_*0-t*_ area under the plasma concentration–time curve from zero to the time of the last measurable concentration; *AUC*_*0-∞*_ area under the plasma concentration–time curve from zero to infinity; *t*_*max*_ time to the first occurrence of *C*_*max*_; *k*_*a*_ absorption rate constant; *k*_*el*_ elimination rate constant; *t*_*0.5*_ half-life in the elimination phase; *Cl/F* apparent plasma drug clearance, *V*_*d*_*/F* apparent volume of distribution

^*^Geometric mean (G_mean_) ratio between the I_MET+OLA_ and II_MET_ groups (%) with a 90% confidence interval (CI) in the brackets. Table 1A was shown *C*_max_, *t*_max_ and Table 1B was shown the AUC- or k_el_-dependent results of analyses

### HPLC–UV assay of metformin

High-performance liquid chromatography (HPLC) with ultraviolet (UV) detection (HPLC Waters 2695 Separations Module with autosampler, Waters 2487 Dual Absorbance Detector) [17] after a liquid–liquid extraction with a mixture of 1-butanol:*n*-heptane (50:50, v/v) was applied to measure the concentrations of metformin in the rats’ plasma. An analytical Symmetry® C8 column (250 × 4.6 mm, 5.0 μm; Waters Corporation, Milford, MA, USA) and a mobile phase consisting of 0.1 M ammonium formate solution, pH 6.3 with an isocratic flow rate of 1.0 ml/min were used. The volume of each injection was 20 µl, and the retention times for metformin and internal standard (acetaminophen) were 3.5 and 11.2 min, respectively.

### UPLC-MS/MS assay

Olaparib in the plasma samples was quantified with an ACQUITY 1 plus ultra-high-performance liquid chromatograph combined with a Xevo TQ-S micro triple quadrupole mass spectrometer (Waters Corporation, Milford, MA, USA) [18]. A Cortecs UPLC C18 column (2.1 × 50 mm, 1.6 µm, Waters Corporation, Milford, MA, USA) was used for chromatographic separation. The column temperature and injection volume were set at 40 °C and 1 µl, respectively. The mobile phase comprised acetonitrile (eluent A) and 2 mM ammonium acetate in water (eluent B) with 0.1% 98–100% formic acid. The flow rate was maintained at 0.3 ml/min. The gradient elution was as follows: 0–3 min, 5% A; 4–5 min, 95%, A; 3–5 min, linear from 5 to 95% A; 5–6 min, linear from 95 to 5% A. The mass spectrometer operated in the multiple reaction monitoring mode. Two transitions for olaparib and olaparib-d4 (IS) were monitored:* m/z* 435.1 → 367.1 and 435.1 → 281.0 (qualifier transition) for olaparib, and* m/z* 439.1 → 367.1 and 439.1 → 281.0 for IS.

## Pharmacokinetic evaluation

The following pharmacokinetic parameters of olaparib and metformin were calculated with the Pkanalix 2023R1 software (Lixoft, France): the elimination rate constant (*k*_e_), the absorption rate constant (*k*_a_), the half-life in the elimination phase (*t*_1/2_), the area under the concentration–time curve from zero to the last measurable concentration (AUC_0–t_), the area under the plasma concentration–time curve from zero to infinity (AUC_0–∞_), the apparent plasma drug clearance (*C*l/*F*), and the apparent volume of distribution (*V*_d_/*F*). The maximum plasma concentration (*C*_max_) and the time to reach the *C*_max_ (*t*_max_) were obtained directly from the measured values. All of the above-mentioned parameters underwent statistical analysis.

## Statistical analysis

The SAS software, version 9.4 (SAS Institute Inc., Cary, NC, USA) was used for statistical analysis. The Shapiro–Wilk test was used to determine the normality. Two pairs of groups were analyzed: I_OLA+MET_ vs. III_OLA_ and I_OLAR+MET_ vs. II_MET_ independently. The differences between the normally distributed variables were determined with the Student’s *t* test. The variables which were not normally distributed were analyzed with the Kruskal–Wallis test at a significance level of *p* < 0.05.

## Results

### Analytical method validation

**Fig. 3:**
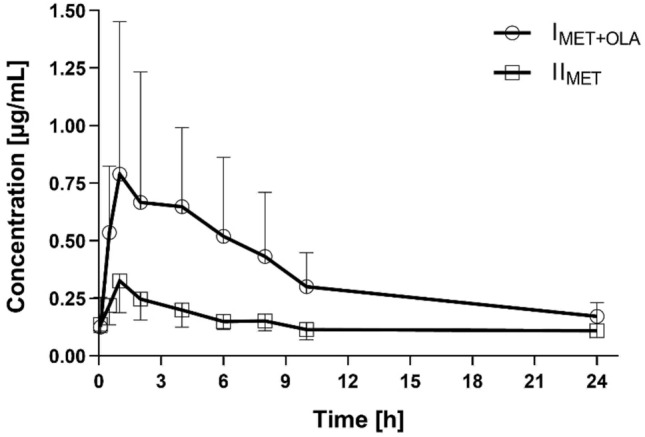
Metformin plasma concentration–time profiles (mean ± SD) in the rats which received metformin (II_MET_) and metformin + olaparib (I_MET+OLA_)

**Fig. 4:**
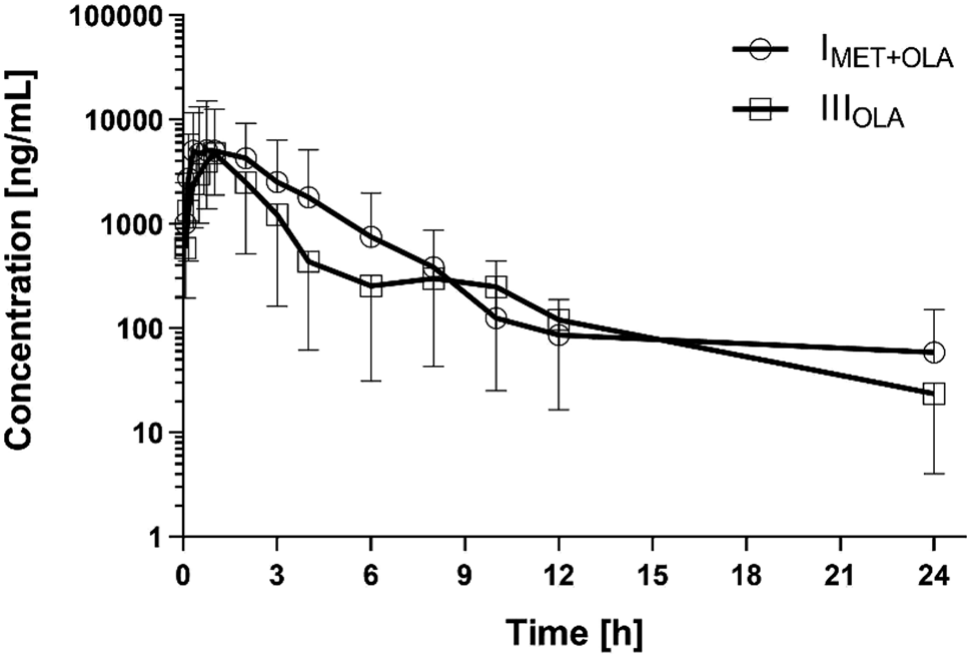
Olaparib plasma concentration–time profiles (mean ± SD) in the rats which received olaparib (III_OLA_) and metformin + olaparib (I_MET+OLA_)

The methods were validated according to the published European Medicines Agency guideline [19]. The calibration curves for metformin were linear (*r* > 0.997) within concentration ranges of 0.1–4.0 µg/ml. Within- and between-run precision (coefficient of variation, CV) and accuracy (%bias) were determined for the following metformin plasma concentrations: 0.1, 0.3, 2.0, and 3.2 µg/ml. The CV was less than 13% and 10%, whereas the accuracy was less than 8% and 5% for all within- and between-run concentrations, respectively. The calibration curves for olaparib were prepared within a range of 10–20,000 ng/ml with a correlation coefficient *r* > 0.99. The lower limit of quantification (LLOQ) was determined at 10 ng/ml with acceptable precision and accuracy and S/*N* > 10. The accuracy, determined as %bias, was ≤ 13% across three quality control (QC) levels and < 20% for the LLOQ. The intra- and inter-run precision of the assay (coefficient of variation) was within 15% for the QC samples and below 20% for the LLOQ. Pkanalix software was used for pharmacokinetic analyses and to analyze the plasma concentration-versus-time data. A non-compartmental model was used to calculate the values of the pharmacokinetic parameters. The results are listed in Tables 1 and 2. Figures 3 and 4 represent the mean plasma concentration–time curves after the oral administration of metformin and olaparib to the rats.

**Table 2:** Plasma pharmacokinetic parameters of olaparib (OLA) after the oral administration of a single dose of olaparib (100 mg/kg b.w.) to the III_OLA_ group and metformin + olaparib (100 mg/kg b.w. + 100 mg/kg b.w.) to the I_MET+OLA_ group

Pharmacokinetic parameters	III_OLA_ (*n* = 8)	I_MET+OLA_ (*n* = 8)	*p* value I_MET+OLA_ vs. III_OLA_	G_mean_ ratio*** (90% CI) I_MET+OLA_ vs. III_OLA_
*C*_max_ (ng/ml)	8716.73 ± 6798.10 (78.0)	9416.94 ± 6330.79 (67.2)	0.8342^*^	1.22 (0.50; 2.99)
AUC_0-t_ (ng × h/ml)	28,276.56 ± 28,159.91 (99.6)	28,253.68 ± 21,387.83 (75.7)	0.7527**	1.38 (0.49; 3.83)
AUC_0-∞_ (ng × h/ml)	28,575.50 ± 28,313.46 (99.1)	29,016.69 ± 21,601.76 (74.4)	0.7527**	1.41 (0.51; 3.90)
*t*_max_ (h)	1.18 ± 0.91 (77.8)	1.11 ± 0.75 (67.7)	0.7087**	0.94 (0.43; 2.07)
*k*_a_ (h^−1^)	0.92 ± 0.29 (32.1)	1.23 ± 0.15 (41.6)	0.1509*	1.28 (0.88; 1.87)
*k*_el_ (h^−1^)	0.44 ± 0.34 (77.5)	0.34 ± 0.22 (65.6)	0.4784*	0.97 (0.41; 2.31)
*t*_0.5_ (h)	4.79 ± 6.06 (126.5)	3.18 ± 2.28 (71.7)	0.7527**	0.97 (0.41; 2.31)
Cl/*F* (l/h)	7.84 ± 15.21 (193.9)	2.62 ± 2.07 (79.0)	0.8336**	0.73 (0.26; 2.03)
*V*_d_/*F* (l)	22.49 ± 24.81 (110.3)	12.51 ± 11.61 (92.8)	0.5995**	0.76 (0.22; 2.62)

Arithmetic means and standard deviations (SD) are shown with coefficients of variation CV (%) in brackets

*C*_*ma*x_ the maximum plasma concentration; *AUC*_*0-t*_ area under the plasma concentration–time curve from zero to the time of the last measurable concentration; *AUC*_*0-∞*_ area under the plasma concentration–time curve from zero to infinity; *t*_*max*_ – time to the first occurrence of *C*_*max*_; *k*_*a*_ absorption rate constant; *k*_*el*_ elimination rate constant; *t*_*0.5*_ half-life in the elimination phase; *Cl/F* apparent plasma drug clearance, *V*_*d*_*/F* apparent volume of distribution

^*^t test for equal variance

^**^Kruskal–Wallis for no normality

^***^Geometric mean (G_mean_) ratio between the I_MET+OLA_ and III_OLA_ groups (%) with a 90% confidence interval (CI) in the brackets

### The influence of olaparib on the pharmacokinetics of metformin

When metformin was co-administered with olaparib, the AUC_0-t_ and AUC_0-∞_ of metformin increased by 159.8% and 74.1%, respectively, as compared with the administration of metformin alone. In the presence of olaparib, the *C*_max_ of metformin increased by 177.8%, whereas the *V*_d_/*F* (65.1%) and Cl/*F* (43.4%) of metformin decreased. However, there were not significant differences between the two groups in the values of the other pharmacokinetic parameters, including *t*_max_ (*p* = 0.0580), *k*_a_ (*p* = 0.1170), and *t*_0.5_ (*p* = 0.0587). There was wide intersubject variability in the pharmacokinetic parameters, as evidenced by the coefficients of variation (CV%) (Table 1: Table 1A was shown Cmax, tmax, and Table 1B was shown the AUC- or kel-dependent results of analysis.). The values of the I_MET+OLA_/II_MET_ ratio for *C*_max_, AUC_0-t_, and AUC_0→∞_ were 2.78, 2.59, and 1.74, respectively.

### The influence of metformin on the pharmacokinetics of olaparib

In comparison with the control group, the V_d_/F (from 22.49 ± 24.81 to 12.51 ± 11.61 l) and Cl/F (from 7.84 ± 15.21 to 2.62 ± 2.07 l/h) of olaparib decreased when it was co-administered with metformin, but there was no statistical significance (*p* = 0.5995 and 0.8336, respectively). There were no significant changes in the other main pharmacokinetic parameters of olaparib. The values of the I_MET+OLA_/II_MET_ ratio for *C*_max_, AUC_0-t_, and AUC_0→∞_ were: 1.08, 0.99, and 1.02, respectively.

## Discussion

As there is a high risk of drug interactions with olaparib, it is important to investigate interactions with other drugs, including metformin, because it is administered to diabetic patients with ovarian cancer [20, 21]. Research has proven that metformin lowers the risk of liver, pancreatic, and breast cancers [21, 22], inhibits the growth of existing cancer cells, and reduces mortality in the course of ovarian, endometrial, and colorectal cancers [23]. Moreover, cell line studies have shown that metformin and olaparib synergistically inhibit tumor growth by blocking the cell cycle [13]. Research on animals assessing the interaction between sorafenib and metformin showed reduced exposure to the anticancer drug, but no changes in the PK parameters of metformin [24]. Moreover, as metformin is believed to inhibit the activation of genes encoding the CYP3A4 enzyme, a significant decrease in the metabolism of substrates (e.g., olaparib [7]) of this enzyme can be expected [25]. It is assumed that interactions occurring at the level of transporters increase the metformin concentration (and thus increase the risk of adverse effects) and decrease exposure to olaparib. This problem is important because it may be necessary to investigate the use of olaparib during the treatment of ovarian cancer and due to the presence of diabetes and the administration of metformin in cancer patients. There have been reports on the increased risk of ovarian cancer among diabetic patients. Diabetes, endometriosis, polycystic ovarian syndrome, as well as several genetic polymorphisms significantly increase the risk of ovarian cancer [26]. Cohort and nested case–control studies conducted by Lee showed that patients with diabetes were at statistically significantly higher risk of ovarian cancer (RR, 1.16; 95% CI, 1.01–1.33), without significant heterogeneity (*I* = 27; *P* = 0.172) [27].

For all these reasons, the pharmacodynamic mechanism of the interaction of metformin with olaparib should be investigated. Additionally, as olaparib raises the blood glucose level by blocking GLUT2 transporters, it results in overestimated fasting glucose level. Therefore, it is very likely that antihyperglycemic treatment will be implemented. It all shows that both drugs can be combined with each other, because type 2 diabetes, to which metformin is dedicated, poses a significant risk of ovarian cancer [7, 10, 11].

### The influence of olaparib on the pharmacokinetics of metformin

There is a high risk of drug interactions with olaparib. In vitro studies have shown that olaparib inhibits BCRP, OATP1B1, OCT1, OCT2, OAT3, MATE1, and MATE2K, which may result from increased exposure to the substrates of these transporters [1]. OCT1, MATE1, and MATE2K are important transporters for the pharmacokinetics of metformin. Metformin is a drug which does not have metabolites. It is excreted in an unchanged form with urine by glomerular filtration and tubular secretion. Metformin only minimally binds to blood proteins. However, it also binds to erythrocytes, which are its second distribution compartment. The role of transporters in the pharmacokinetics of metformin is very complex. OCT1 in enterocytes may influence the transport of metformin into the interstitial fluid. Additionally, the hepatic uptake is also mediated by OCT1. Therefore, the inhibition of OCT1 may decrease the effect of metformin. OCT2 is involved in the uptake of metformin from the blood into the kidney. MATE1 and MATE2K (efflux transporters) are responsible for the elimination of metformin from renal cells to the urine [28–30]. Additionally, the interaction with OCT2 in proximal tubule epithelial cells may increase the systemic disposition of metformin by reduced renal clearance. In our study, the co-administration of a single dose of olaparib with metformin significantly decreased the metformin clearance from 6.31 ± 1.48 l/h to 3.57 ± 1.26 l/h (*p* = 0.0014). The concomitant application of olaparib and metformin increased the metformin *C*_max_ 2.8 times and its AUC 2.6 times. Such an increase in exposure to metformin may cause the risk of side effects, particularly in the gastrointestinal tract. According to some researchers, the weakening of the OCT1 function or the reduction of drug transport through OCT1 may result in gastrointestinal intolerance due to increased metformin concentration in the intestine [31, 32]. It is known that the gastrointestinal distress of metformin seems to be locally driven, hence it is hard to rationalize how do the higher systemic concentrations cause gastrointestinal side effects. Also, there is no clear evidence that bile excretion component of metformin increases in presence of olaparib. However, inhibition of the activity of the OCT1 transporter is an important issue.

Therefore, the effect of olaparib, which is an OCT1 and OCT2 inhibitor, on the increased risk of GI intolerance of patients taking metformin cannot excluded.

It is noteworthy that in the absence of hypersensitivity to metformin, it is used as a first-choice drug in the treatment of type 2 diabetes. Additionally, metformin has been proved to reduce the risk of liver, pancreatic, and breast cancers [21, 22], inhibit the growth of existing cancer cells, and reduce mortality in the course of ovarian, endometrial, and colorectal cancers [23]. Moreover, cell line studies have shown that metformin and olaparib synergistically inhibit tumor growth by blocking the cell cycle [13]. Therefore, metformin is a promising drug in the treatment of cancer.

### The influence of metformin on the pharmacokinetics of olaparib

Metformin is not expected to be involved in many drug–drug interactions (DDIs) but there are studies showing that it has the potential to be the perpetrator in DDIs. Metformin reduced the *C*_max_ and AUC_24_ of aliskiren but the changes were not significant, so clinical DDIs are not expected. There have been studies showing that metformin affects phenprocoumon and warfarin. It is known that the *C*_max_ and AUC_24_ of trospium decreased when it was combined with metformin – probably metformin can inhibit the oral absorption of the drug. On the other hand, metformin was found to increase the exposure to topiramate [33]. Vuu et al. [34] observed that the co-administration of metformin and sotorasib did not affect the sotorasib exposure to a clinically significant extent. It also did not affect the hypoglycemic effect of metformin, although it was different from the one observed in vitro and its duration was shorter.

Chinese researchers hypothesized that the combination of sorafenib and metformin may have a synergistic effect in the treatment of colorectal cancer while reducing the severity of side effects [35]. However, when vandetanib is combined with metformin, the latter may require additional monitoring and periodic dose escalation [36]. The authors of the METAL (METformin in Advanced Lung Cancer) study [37], which was a phase I-II trial, hypothesized that the administration of metformin to non-diabetic patients may revert resistance to gefitinib, which is a selective epidermal growth factor receptor (EGFR) and tyrosine kinase inhibitor applied in non-small cell lung cancer. The researchers observed that a stable blood glucose level was maintained in the non-diabetic population. At the same time, during the 30-week observation period the neoplastic disease became stabilized in 50% of the patients. The combination of metformin with gefitinib inhibits cell proliferation and induces apoptosis, particularly in cell lines harboring the wild-type LKB1 gene. This dependence can also be observed in another tyrosine kinase inhibitor – erlotinib. The time-to-progression median was 20 weeks. This effect may have been caused by the fact that metformin may activate AMP-activated protein kinase and thus inhibit the mTOR and block the MAPK signaling. The relationships between metformin and tyrosine kinase inhibitor are constantly being investigated [37].

Clinical trials on metformin have not shown any influence of this drug on the efficacy of the following medications: alogliptin, dapagliflozin, dutogliptin, gemigliptin, linagliptin, lobeglitazone, rosiglitazone, rosuvastatin, saxagliptin, sitagliptin, and vildagliptin [33]. As metformin is believed to inhibit the activation of the genes encoding the CYP3A4 enzyme, a significant decrease in the metabolism of substrates (e.g., olaparib [7]) of this enzyme can be expected [25]. Gralewska et al. found that the treatment with olaparib and metformin increased oxidative stress and decreased the mitochondrial membrane potential. The co-administration of metformin and olaparib may result in almost two times greater early apoptosis than when the drugs are administered individually. After the co-administration of olaparib with metformin the percentage of late apoptotic cells was significantly higher than when the drugs were given separately (28.4% for co-administration vs. 5.1% for olaparib and 8.2% for metformin) [6]. Another study showed that biguanides in combination with PARP inhibitors synergistically reduced the epithelial-mesenchymal transition, proliferation, and survival of ovarian drug-resistant cancer cells [38].

Our research showed that a single dose of metformin did not have inhibitory effect on olaparib and did not affect its PK parameters. However, olaparib significantly changed the pharmacokinetics of metformin. The *C*_max_ of metformin increased by 177.8%, whereas the *V*_d_/*F* and Cl/*F* of metformin decreased. There were no significant differences between the two groups (metformin co-administered with olaparib and metformin administered alone) in the other pharmacokinetic parameters, including *t*_max_ (*p* = 0.0580), *k*_a_ (*p* = 0.1170), and t_0.5_ (*p* = 0.0587. The values of the I_MET+OLA_/II_MET_ ratio for *C*_max_, AUC_0-t_, and AUC_0→∞_ were 2.78, 2.59, and 1.74, respectively. Investigations in human in vitro systems indicated phase I metabolism of olaparib was CYP mediated and that CYP3A4 and 3A5 were the dominant metabolic enzymes. As expression of CYPs 3A4 and 3A5 is highly variable in human and olaparib clearance in human was primarily metabolic, this may explain some of the variability observed in clinical pharmacokinetics. Perhaps, in the study, the high variability contributed to the lack of statistically significant differences in the PK parameters of olaparib.

There were some limitations to our study, such as the small size of the sample, which was limited by the Local Ethics Committee (No. 45/2022, of 27 May 2022). The lack of using a model is also a significant limitation of the study. Another limitation was the fact that both drugs (not only metformin but also olaparib) were administered only once and at the same dose. If the experiment had been continued to the steady state (as in patients), there might have been changes in the olaparib PK as well. If the experiment had been conducted on pre-diabetic or diabetic animals, the effect of the pathological condition on the PK of both drugs might also have been observed.

## Conclusions

In conclusion, we showed that metformin had no effect on the pharmacokinetics and metabolism of olaparib, but olaparib significantly increased the body’s exposure to metformin, which may be of significant clinical relevance and may be associated with the risk of adverse effects. The presented results require confirmation in a clinical trial.

### Supplementary Information

Below is the link to the electronic supplementary material.

Supplementary file1 (XLSX 23 KB)

## Data Availability

The original contributions presented in the study are included in the article; further inquiries can be directed to the corresponding author.
